# Engaging Hospital Staff to Identify Levers for Adoption of Clinical Decision Support: Protocol for a Single-Site Case Study Using System Dynamics Group Model Building

**DOI:** 10.2196/80848

**Published:** 2026-04-21

**Authors:** Nina Rachel Sperber, Lindsey Eileen Zimmerman, Lauren Caton, Tom Rust, Armando Bedoya, Sarah Haas, Lori Orlando, Ben Goldstein

**Affiliations:** 1 Department of Population Health Sciences, Duke University School of Medicine, 215 Morris St., Suite 210, Durham, NC, 27701, United States, 1 919-681-6801; 2 Durham VA Health Care System, Durham, NC, United States; 3 VA Palo Alto Health Care System, Dissemination and Training Division, National Center for PTSD, Menlo Park, CA, United States; 4 Division of Public Mental Health and Population Sciences, Department of Psychiatry and Behavioral Sciences, Stanford University School of Medicine, Palo Alto, CA, United States; 5 University of North Carolina, Chapel Hill, NC, United States; 6 United States Department of Veterans Affairs, Washington, DC, United States; 7 Department of Biostatistics and Bioinformatics, Duke University School of Medicine, Durham, NC, United States; 8 Department of General Internal Medicine, Wake Forest University School of Medicine, Winston Salem, NC, United States

**Keywords:** clinical decision support, machine learning, early warning score, system dynamics, group model building, implementation science

## Abstract

**Background:**

Clinical decision support (CDS) tools that provide patient-specific and evidence-based information to clinicians and care managers regarding patient risk for adverse outcomes have been a part of health care for decades. However, modern CDS, which consists of automated predictions based on complex machine learning models and hundreds of complex input variables, faces obstacles to adoption related to health care providers’ perceptions of lack of transparency and utility. Often, the expertise of data scientists and clinical end users is not well integrated, creating implementation gaps from CDS development to adoption and ongoing implementation.

**Objective:**

This protocol describes the use of group model building (GMB) from the field of system dynamics to engage health system staff in identifying dynamic facilitators and barriers to implementing 1 class of CDS—early warning scores (EWSs)—in general medical-surgical wards. We aim to produce a causal model that reflects the insights and feedback shared during these sessions. We will also evaluate the GMB process as a potential strategy for CDS implementation and adoption more generally.

**Methods:**

The protocol consists of 3 sequential GMB sessions designed to elicit key variables for inclusion in the model, understand how changes in variable behavior over time affect adoption, and develop a causal loop diagram. Pre- and postsession questionnaires assess changes in perceived acceptability, appropriateness, and feasibility of the EWS and collect feedback on the GMB process. A stock-and-flow simulation model will be developed from the causal loop diagram to quantify how feedback loops influence variables over time and test assumptions.

**Results:**

The project was funded in 2022-2025, and 3 GMB sessions and qualitative causal loop diagrams were completed during that time. Data analysis is ongoing. This analysis consists of translating the causal loop diagram from the GMB into a stock-and-flow simulation model to quantify how feedback loops influence variables over time. Results will include a causal loop diagram accompanied by a detailed narrative that together tell a story about system behavior surrounding EWS adoption that is supported by session transcripts, the simulation model and test results, and the data on GMB participants’ views about the EWS itself and the modeling process.

**Conclusions:**

These findings will have broader applicability beyond just EWSs. Future work will build on the EWS system dynamics model by incorporating multiple clinical use cases to fully capture multilevel factors that determine real-world adoption and sustainability of machine learning CDS.

## Introduction

### Problem

Clinical decision support (CDS) tools provide patient-specific and evidence-based information to health care providers and care managers regarding patient risk for adverse outcomes. CDS has been a part of health care for decades and is a key component of the learning health care system [1]. Overall, CDS has led to improvements in patient and health care provider outcomes [1-3]. With the advent of integrated electronic health record (EHR) systems over the past decade, there has been a proliferation of predictive models, that is, algorithms based on clinical, typically EHR, data that generate estimates of the probability of outcomes (eg, sepsis, readmission, and mortality) for any given patient, and in turn can alert health care providers to the need for specific interventions [3,4]. Predictive models can use a variety of approaches to compute algorithms ranging from simple logic-based rules to interpretable statistical models to, more recently, machine learning (ML) algorithms that learn from examples and include the use of both structured and unstructured data [5].

While predictive ML models of any type can improve the identification of high-risk patients, there are obstacles to implementation and adoption. Clinicians have responded to a perceived overabundance of CDS alerts by developing “alert fatigue” and ignoring the CDS [6,7]. In fact, prior research indicates that between 49% and 96% of alerts are ignored or overridden [8-10]. Nonresponse to alerts often occurs when clinical users do not understand or trust the CDS [11,12]. ML-based CDS faces particular obstacles to adoption by users who perceive a lack of transparency in how predictions are made, affecting their views about its validity or usefulness in clinical care [9,13]. Participatory approaches that include input from health system staff and others throughout the CDS lifecycle, from algorithm development to implementation and maintenance, could improve the adoption of ML-based CDS [14]. Typically, only a few select clinical users contribute to the developmental process, and data scientists are often not well integrated within the clinical environment, even though their design decisions impact downstream use of the CDS tools [15]. This lack of integrated participation creates a barrier in communication and knowledge transfer between the technical and clinical realms, thereby limiting the utility and application of CDS in routine clinical practice.

### Objective

The objective of this project is to bring health system staff together to identify facilitators and barriers to ML CDS implementation in their contexts. To do this, we use group model building (GMB), a methodology from the field of system dynamics that facilitates collaborative modeling of complex problems [16,17]. In system dynamics, complex processes are modeled by specifying variables, feedback loops, and time delays. In GMB, accuracy, impact, and usability of system dynamics models are enhanced by directly involving individuals with lived experiences of the problem being addressed to develop the models [18]. Used most commonly in organizational change management as a strategic planning technique to identify solutions from the bottom-up, GMB has more recently emerged as a recommended strategy to address problems with adoption of evidence-based practices for health care [19,20]. In this case, GMB is used to foster a shared understanding of why the implementation problem persists over time and to identify potential solutions as compared to the status quo [16]. Our research uses GMB to engage a multidisciplinary group of health system staff involved in CDS implementation.

## Methods

### Study Design

This protocol uses a single-site case study design within a single health care system. GMB is used within the case study to elicit and represent stakeholders’ perspectives on adoption dynamics. Together, this design and methodology enable an in-depth exploration of conditions influencing adoption of ML-enabled CDS in a real-world implementation setting.

### Use Case

This case study focuses on 1 class of CDS, early warning scores (EWSs), which have been adopted well in some settings but not others. EWSs are widely used by both large academic and small community hospitals to identify patients at risk for decompensation, leading to intensive care or mortality [21]. Using predominantly vital signs, EWSs typically predict the need for an intensive care unit transfer and/or the risk of death within a 12- to 48-hour time horizon, typically referred to as unexpected decompensation. The first widely used EWS was the National Early Warning Score (NEWS), a point-based score of 7 vital signs measurements [22]. Since the introduction of the NEWS, over 20 EWSs have been introduced in the literature, ranging from simple modifications of the NEWS (eg, extra laboratory measurement) to more complex scores that integrate laboratory data, demographics, and comorbidities [23]. Over the last several years, EWS scores have evolved from point-based scores to logistic regression–based scores (with variable weights) to complicated ML-based scores. Benefits of EWS adoption have been observed. For example, a large analysis across Kaiser Health indicated a 16% (10%‐22%) reduction in the adjusted relative risk of mortality in hospitals that used their EWS [24]. As a result of findings such as these, investments are continually made to develop and improve EWS CDS, and there are many choices involved in EWS implementation. These range from the target user (doctor or nurse), type of model (ML or points based), and temporality of the alert (real time or interval based). Our prior research showed that due to lack of adoption, the technical integration of NEWS into the EHR of study sites did not change rates of mortality or unanticipated intensive care unit transfer, even when the accuracy of the ML score indicated it was performing well [12]. Frontline nurses in our health care system ignored the alert 86% of the time [12]. In discussion groups, nurses expressed neither understanding of how the score operates nor how to use it clinically and described experiences with unhelpful CDS behaviors, including reports of alerts that were not accepted continuing to fire on the same patient. The EWS developers subsequently amended the score to present a more qualitative—and easily interpreted—assessment of risk and to fire only around shift changes (8 AM and 8 PM) as opposed to continually throughout the day [25].

### Setting

This study leverages Duke University Health System’s (DUHSs) broad experience with EWS, often talked about monolithically despite existing within a variety of contexts. Within the DUHS, different EWS implementation contexts create a natural variation. This variation allows us to assess how different conditions affect EWS adoption.

We selected 2 hospitals within the DUHS, a large quaternary care hospital, Duke University Hospital (DUH), and a smaller community hospital, Durham Regional Hospital (DRH), that use 2 different EWS types. While the hospitals have used a shared DUHS Epic-based EHR system since 2013, they also have separate governance structures as to how and which CDS tools are implemented. In 2015, DUHS implemented the NEWS score across 3 hospitals. After the impact evaluation in 2019, the score was turned off at DUH and replaced with a homegrown EWS trained on DUH data [12]. This resulted in the continued use of the NEWS at DRH and the use of Duke EWS at DUH, providing an opportunity to compare conditions that affect adoption [25].

The NEWS and Duke EWS differ in 5 specific ways. First, the underlying scores are fundamentally different with regard to the input variables and the information that the score will display. The NEWS is a points-based score consisting of 7 input variables. Users can see both a patient’s overall score as well as the points associated with each of the input variables. Conversely, the Duke EWS is an ML-based score consisting of over 40 input variables. As such, it is not possible to discern how any 1 variable impacts a patient’s overall score. Second, while both scores are directly integrated into the EHR, they are operationalized differently in terms of how they are integrated into the workflow with timing of alerts. The NEWS is operationalized as a continuously firing alert that provides real-time feedback. The Duke EWS, based on user input, is designed to be used at shift change only (ie, discrete time points), to inform which patients are most critical. Third, each score has different target users. The NEWS broadly targets staff nurses caring for admitted patients, while the Duke EWS targets primarily charge nurses, responsible for overseeing staff and operations of the staff nurses. Fourth, the scores differ in the information displayed. The NEWS is presented to the end user as an overall score. The Duke EWS presents as a color-coded 3-level risk category (red, yellow, and green). Fifth, overall model interpretability differs. The overall score that the NEWS displays has no specific probabilistic interpretation. The alert fires when the score crosses a prespecified threshold. However, Duke EWS risk categories indicate a patient’s probabilistic risk level. The risk category thresholds were chosen based on desired operational performance with respect to sensitivity and positive predictive value.

### Participant Recruitment

We identified developers (data scientists), implementers, and users who had interacted with 1 of the 2 EWS versions in use at DUHS through snowball sampling, asking an initial contact identified by a member of our study team to refer us to other interested stakeholders. They were invited to participate with an emailed letter signed by the principal investigators. We continued in this manner until we recruited at least 1 developer and implementer for each or both of the 2 EWS scores and at least 2 users of each score. Everyone was asked to participate in 3 sessions. To minimize attrition, we scheduled the 3 sessions as a series at 1 time and according to the participants’ availability and kept each session to a maximum of 2 hours.

### Ethical Considerations

This study was reviewed and approved by the DUHS Institutional Review Board (Pro00111061). All participants provided informed consent prior to participation. Participants who consented and attended 1 or more GMB sessions were included in the study, with attendance across all 3 sessions encouraged but not required. Participant privacy is protected through use of unique study ID numbers, secure storage of reidentification logs in restricted‑access Duke Box and network drives, deidentification of all materials, aggregate reporting, and destruction of audio recordings after publication; no identifiable names are required during group sessions or recordings. Participants received no compensation.

### GMB Overview

#### Overview

The 3 sessions were designed to engage hospital staff in developing a model of EWS adoption using the established GMB activities of variable elicitation (to identify key variables for inclusion in the model), graphing behavior over time (to understand how change in variable behavior affects adoption), and causal loop diagramming (to visualize how feedback loops affect adoption) [26-28]. Each session was built on the outputs of the previous one. These sessions were delivered using the Zoom application (Zoom Communications, Inc) with a password entry, audio recording, and transcriptions generated. Miro electronic whiteboard (Miro, Inc), a collaborative platform, was used as a group workspace [29,30]. The core modeling team planned and participated in all sessions, with each person having a distinct perspective and role: the team included a coordinator who organized and managed the flow of the sessions; a trained GMB facilitator who conducted the sessions; a qualitative researcher who served as a cofacilitator; a biostatistician or data scientist with experience developing CDS ML algorithms who observed as a subject matter expert; and a clinician data scientist who provided a community-based perspective. A detailed facilitation guide specified timing, roles, and scripts (Multimedia Appendix 1).

#### Session 1: Variable Elicitation

The first session used variable elicitation to identify key variables affecting EWS adoption in general medicine settings for inclusion in the system dynamics model [26]. This session included 10 staff who worked with NEWS or Duke EWS. They were brought together in 1 session with the end goal of converging on a set of key variables. It began with the facilitator presenting the problem of failure to use the EWS CDS, showing a depiction of the CDS development-to-use gap in the form of a conceptually stylized graph with a line representing the hoped-for trajectory of a high, steady level of commitment compared to the observed trajectory of declining commitment. This type of image is used in GMB to define the problem with the group and create a shared reference [31]. The facilitator asked the group whether the image captured their perspectives and if changes were needed. The facilitator then asked participants to describe components that influence the current trajectory with questions about perceived acceptability (What are key factors that inhibited or facilitated satisfaction with EWS?), perceived appropriateness (What are key factors that inhibited or facilitated compatibility with practice settings or users?), and perceived feasibility (What are factors that inhibited or facilitated successful use of the EWS in their setting?). The cofacilitator posted responses on the whiteboard in thematic clusters. The facilitator probed for detail, such as whether the clusters resonated with the group, whether other themes should be included, or if themes should be removed, and asked participants to prioritize factors in order of importance.

#### Session 2: Behavior Over Time

The second session aimed to understand how the variables identified in the first session changed or could change over time [27]. In this session, the facilitator asked participants to graph trajectories of the variables on the whiteboard, with the x-axis indicating progression of time and the y-axis indicating changing values of the concept. The graphs included hoped-for, expected (“business as usual”), or feared trajectories. The facilitator probed participants to discuss their hypotheses about what causes and is caused by these trajectories. For example, participants identified quality of data input as a factor affecting use of the score, and the facilitator asked them to describe more about how they had observed the quality of inputs changing during a patient’s stay in the unit. The DUH staff, DRH staff, and developers met separately from each other to facilitate identification of themes specific to their contexts.

#### Session 3: Causal Loop Diagrams

The third session engaged all staff to converge on a hypothesis about structural drivers of EWS adoption, that is, the underlying factors and relationships that shape how the EWS is used in practice, by contributing to the development of a causal loop diagram, which visually depicts these relationships through feedback loops [28]. Prior to the session, the study team developed a simple causal loop diagram to provide an initial scaffold of causal connections for participants to review, critique, and elaborate by reviewing notes from the prior sessions, “walking through” causal relationships and loops, and considering how the diagram fit with what they knew about the behavior of hospital units. The facilitator initiated the GMB session by reminding the group of the problem and the variables and graphs discussed in the earlier exercises. The facilitator then presented the causal loop diagram and solicited input by asking participants whether the diagram reflected their experiences, if they had any changes, and whether the directionality of relationships between variables depicted (eg, increase in one variable causes an increase in another vs an increase in one variable causes a decrease in another) was accurate. After this session, the study team created a new version of the causal loop diagram according to content from the session and their own observations of the health system. The team met with health system staff to validate this synthesis through an individual interview with a charge nurse who had participated in the sessions and a group interview with 2 staff from in-hospital and nursing leadership who worked with EWSs and had not participated in the sessions.

### Measures

A questionnaire administered at 4 time points, prior to the first session and following each session, measured changes in participants’ views of the EWS over the course of their involvement with GMB. Validated implementation science measures of perceived acceptability of an intervention (whether they like it), perceived appropriateness of an intervention (whether it fits with their practice), and perceived feasibility of an intervention (whether they can successfully use it) were used [32]. These measures were selected because collectively the constructs can act as a proxy for assessing commitment to adopting a new tool such as the EWS [32]. After each session, participants also provided feedback on the GMB process (Multimedia Appendix 2).

### Analysis

#### System Dynamics Modeling

This analysis consists of integrating quantitative and qualitative information. The GMB causal loop diagram and transcripts will serve as the primary qualitative data sources. We are translating the causal loop diagram from GMB into a stock-and-flow simulation model to quantify how feedback loops influence variables over time. In a stock-and-flow model, stocks represent quantities that accumulate or decrease, such as patient counts, while flows indicate the rates at which these stocks change. The stock-and-flow simulation model will reflect the insights and feedback shared during the GMB sessions, with adjustable parameters to explore what would happen to system behavior if 1 or more values change. We will define each stock and flow relationship with mathematical equations and capture delays, nonlinearities, and feedback effects using Vensim (Ventana Systems) simulation software’s built-in functions. We anticipate generating time-series graphs, which show how key variables, such as patient counts or adoption of CDS, change over time. We will also conduct scenario comparisons, running the model under different assumptions to see how outcomes vary. The research team will thus use the simulation model to test the internal logic of the causal loop diagram and refine its underlying assumptions.

#### Statistical Analysis

The statistical analysis will consist of descriptive summaries of presession and postsession questionnaire data to compare responses by developers and the DRH and DUH settings. We will calculate the mean, SD, and range of the Acceptability of Intervention Measure, Intervention Appropriateness Measure, and Feasibility of Intervention Measure and the feedback items for each group [32]. We will construct a dot plot using RStudio for the Acceptability of Intervention Measure, Intervention Appropriateness Measure, and Feasibility of Intervention Measure that shows the mean score for each of the 3 groups at the 4 time points when the questionnaire was administered (prior to the first session and after each of the 3 sessions). We will also construct a dot plot that shows the mean score for each of the feedback items after each of the 3 sessions along with the open-ended responses.

## Results

The project was funded in 2022-2025, and three GMB sessions were completed during that time. Data analysis is ongoing. Results will include a causal loop diagram accompanied by a detailed narrative that together tell a story about system behavior surrounding EWS adoption. The causal loop diagram will be supported by session transcripts, the simulation model and test results, and the data on GMB participants’ views about the EWS itself and the modeling process. These results will illustrate processes and mechanisms that affect EWS adoption under complex, dynamic real-world conditions.

## Discussion

The results will have broader applicability beyond just EWSs. When developing and implementing ML-enabled CDS tools in general, many decisions need to be made. These include the nature of the underlying score, who the user base is, and how and when it is presented to those users. Often, these decisions stem from siloed and artificial test environments that limit the ability to evaluate how they function in real-world settings. Early in the model building process, developers often make design decisions that impact the overall implementation of the CDS, typically with usability studies of single tools conducted with individual user groups in research settings [33,34]. Although these studies demonstrate technical capability of the tools, a major limitation is that they do not consider applied function within the context of typical clinical care workflows. Once the CDS is embedded in the clinical setting, there is often a disconnect between what the developer intended and users’ interaction. Such an orientation is likely to cause low adoption by end users. This approach to engage developers, implementers, and users to create a shared visual model during implementation planning or execution will help to improve their understanding of the problem that the CDS is meant to address and, in turn, improve buy-in and the potential for adoption [17,35].

Future work will build on the EWS system dynamics model that is the focus of this protocol by incorporating multiple clinical use cases to fully capture multilevel factors that determine real-world adoption and sustainability of artificial intelligence (AI)-CDS. The resulting interactive simulation model will provide an environment to test the implementation of AI-enabled CDS more generally across health systems and identify intervenable levers to improve efficiency, reducing the reliance on costly real-world research usually needed to identify implementation barriers, facilitators, and strategies. The simulation model will provide an interactive learning infrastructure for responsible AI deployment to understand the behavior of AI tools as they move from development to implementation by allowing users to visualize effects over time.

Interactively modeling system dynamics through GMB and a simulation model promotes a learning health care system environment by allowing those who develop, implement, and use CDS tools to reflect on what did and did not work in that process [36]. This approach could offer value during the exploratory phase, when decision-making occurs, to better understand implementation issues and help define implementation strategies that consider not only CDS features but also system complexity, and thus improve sustainability of ML-enabled CDS over time [37]. Having a way to support the ongoing planning and monitoring of any ML-enabled CDS in health care is important to ensure its safe and effective use given an evolving regulatory framework, and given that most ML-enabled tools depend on nuances of local data [33]. This approach to understanding facilitators and barriers to EWS adoption will be useful for researchers and practitioners interested in proposing an informatics implementation project.

## Supplementary material

10.2196/80848 Multimedia Appendix 1 Facilitation guide.

10.2196/80848 Multimedia Appendix 2 Questionnaire for feedback.

10.2196/80848 Multimedia Appendix 3 Artificial intelligence prompts.

## Abbreviations

AI: artificial intelligence
CDS: clinical decision support
DRH: Duke Regional Hospital
DUH: Duke University Hospital
DUHS: Duke University Health System
EHR: electronic health record
EWS: early warning score
GMB: group model building
ML: machine learning
NEWS: National Early Warning Score
