# Supplementary material for: Engaging Hospital Staff to Identify Levers for Adoption of Clinical Decision Support: Protocol for a Single-Site Case Study Using System Dynamics Group Model Building
Source: JMIR Res Protoc. 2026 Apr 21;15:e80848. doi: 10.2196/80848 (PMC13099119; doi:10.2196/80848)
Supplement: Multimedia Appendix 1 [file resprot-v15-e80848-s001.docx]

­­

**Early Warning Score Implementation Group Model Building**

**Facilitation Manual**

*Last Updated: 10/22/2023*

Acknowledgements

The following facilitation manual was based on instructors Kelsey Werner and Ellis Ballard for the Group Model Building track of the Systems Science for Social Impact (SSSI) 2022 Summer institute in St. Louis, MO hosted 25-29 July 2022.

Materials in this facilitation manual are adapted from resources of Scriptapedia (<https://en.wikibooks.org/wiki/Scriptapedia>) are made available under Creative Commons Licensing for use & adaptation by system dynamics practitioners & researchers.

This facilitation manual is shared as a learning resource and as an artifact of this course. It is licensed as well under Creative Commons Share Alike licensing, which allows reusers to distribute, remix, adapt, and build upon the material in any medium or format, so long as attribution is given to the creator. The license allows for commercial use. If you remix, adapt, or build upon the material, you must license the modified material under identical terms.


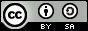


**Attribution-ShareAlike**

**CC BY-SA**

# Group Model Building Objectives

## Explicit Objectives

- Reveal differences in mental models about dynamics of Early Warning Score (EWS) implementation
- Reveal commonalities in mental models about dynamics of EWS implementation
- Provoke a feedback systems perspective on AI-enable clinical decision tools

## Implicit Objectives

- Bring EWS developers, users, owners, and implementers together to co-define common barriers and facilitators to key implementation outcomes (perceived acceptability, appropriateness, feasibility)
- Evaluate effect of GMB sessions on changes in perceived acceptability, appropriateness, and feasibility of EWS (commitment to adopt)

# Session 1: Problem Definition & Factor Elicitation

# Summary Agenda

## Agenda

| **Time** | **Activity** |
| --- | --- |
| 7:45-8:00 | Team members join call |
| 8:00-8:05 | Participants enter Zoom |
| 8:05-8:10 | Opening: Walk Through Tasks |
| 8:10-8:30 | Presenting the Reference Mode |
| 8:30-9:15 | Task Ask - Miro |
| 9:15-9:30 | Share out Variable Elicitation by Theme |
| 9:30-9:45 | Close |

# Detailed Agenda – Session 1: Variable Elicitation

| **Time** | **Lead(s)** | **Activity** |
| --- | --- | --- |
|  | Community Facilitator: | **Set-up**   - Use Teams channel for backchannel communication and ensure that everyone has mobile app installed and working. - Preload Miro sticky notes   - Barriers/Facilitators and black sticky notes grouped by the three outcomes - Send Zoom instructions (joining via computer not phone, etc.) in email reminder - All team members download Teams app, download all documents to hard drive - Email slide deck ahead of time and tell participants that they can print out to have on hand if they want. - Facilitators open Miro on their computers. |
| 7:45am | All | *Team members join call*   - Open necessary materials (for viewing and presenting), login to Miro & navigate to board |
| 8:00am | Community Facilitator: | **As people enter the Zoom**  Ask participants to change their Zoom labels to their first name and their institution (DUR or DRH). We will also share the slide deck in the chat – please click the file to download/ then open to reference during today’s session.  *Post below instructions and share slide deck document in the chat****.***   - Hi and welcome! As you settle in, please update your Zoom label to your first name and your institution (DUR, DRH, SOM or previous affiliation at Duke). You can update this by clicking the 3 dots in the top right corner of your Zoom square then selecting ‘Rename’ at the bottom of the list. - Share slide deck document   Once everyone has entered Zoom: Hi everyone, thank you so much for joining. Before we begin, I wanted to let you know that we are starting our recording of today’s session now. Thank you.  ***Community Facilitator starts recording*** |
| 5 minutes  8:05-10 am | Convener:  Community Facilitator:  Timekeeper: | **Opening: Walk Through Tasks**  *Timekeeper: keep time and send alerts in Teams chat*  Convener: Welcome.    We have invited you here, because you all interact or have interacted with the Early Warning Score (or EWS) at Duke Health, and we want to learn from you about your experiences.  *Community Facilitator shares first slide.*  If we were to ask each of you to describe how the EWS affects patient care at Duke Health, you would likely have different stories. Each of us has our own valid mental picture of the parts, but our understanding of the whole is limited by our own unique vantage point. So, if we want to understand how EWS works system-wide, we need to include and integrate different perspectives. Ultimately, we want to hear from everyone and then converge on a shared understanding.    Today we will start by sharing our understanding of a general problem with EWS use and then invite your feedback. We will then build off of that discussion to understand facilitators and barriers. This work will help to plan for future implementation of these kinds of scores.    Before we do that, I would like to briefly introduce our team.  *Community Facilitator advances to second slide (Team).*  Now, we’d like to share some ground rules to help us in our discussion today. We will post these as well in the chat for your reference.    *The Community Facilitator posts instructions for introductions and ground rules in Chat while Convener is speaking.*   - When speaking for the first time, please introduce yourself briefly (Name, role and location at Duke - current or former) - DUR, DRH, SOM or previous affiliation at Duke - In addition to speaking, we encourage you to use the chat to share thoughts or ask questions - Please jot ideas and thoughts down as you have them (before sharing verbally or in the chat) so you don’t lose your thought - We want to hear from everyone, so please share throughout the discussion today and step back to let others speak. - Everyone’s perspective is very important and we recognize that not everyone is comfortable speaking in group settings. To ensure that we hear from all, we may call you in if we haven’t heard from you yet.     *Convener pauses to ask if anyone has any questions.* |
| 8:10-30  20 minutes | Convener:  Modeler Facilitator:  Community Facilitator:  Reflectors: | **Presenting the Reference Mode**  *Community Facilitator advances to third slide (Reference Mode).*  Implementing ML-enabled CDS  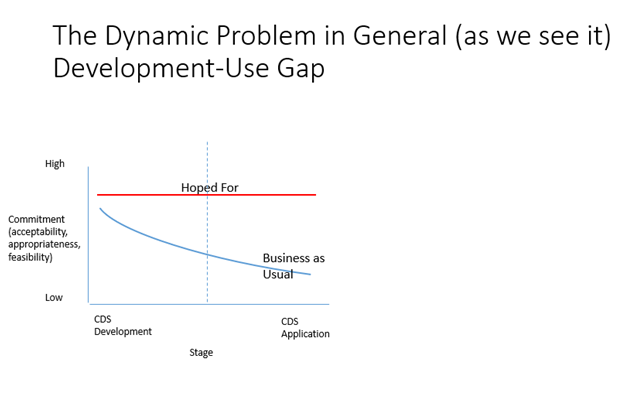  Modeler Facilitator: This graph represents what we know about the general problem of EWS adoption. We are focusing on the concept of commitment to adopt. Do people want to use it and believe that they can? This graph shows that commitment is high during the development stage (blue line) and then drops when applied in real-world settings. The red line represents our hoped for trajectory, that commitment is maintained over time. This understanding of the problem will anchor our understanding in today’s and future sessions.  We will spend the next 15 minutes negotiating: Does this capture your perspective? Is there anything you would change?’  *Reflectors contact Model Facilitator by Teams chat to direct understanding and probing.*  *[Keep up reference mode slide while participants discuss]*  This was great feedback; we’re going to take what we learned here into the next section  *Take screenshot of Zoom whiteboard with reference mode*  *[Cue to pause sharing, close slides and open up Miro]* |
| 8:30-9:15  45 minutes | Community Facilitator:    Modeler Facilitator :  Theme Builder:  Reflectors: | *Community Facilitator closes out slides and opens Miro to #1 Satisfaction with EWS and Themes (centered on both, only show #1 at start, 50% Zoom); will monitor chat and share participant responses verbally.*  *Theme builder to add notes that people say to Miro board as needed*  **Tech notes**  *Move around Miro with right click button on mouse. Use bar on far right to scroll down.*  *Ctrl+D to duplicate post it notes as needed.*  **Task Ask**  Model Facilitator*:*  Keeping in mind the trajectory we had earlier, we want to discuss the components that influence the current trajectory, and what facilitators could promote the desired trajectory.  We have three components; we will have 15 minutes for each component. Please keep responses short, 1-3 words if possible. Meant to be rapid fire. Also step up & step back (explain).   - What are key factors that inhibited satisfaction with EWS? What are key factors that facilitated satisfaction with EWS? [Response time 7 minutes each] - What are key factors that inhibited compatibility with practice settings or users? What are key factors that facilitated compatibility with practice settings or users? [Response time 7 minutes each] - What are key factors that inhibited EWS’ successful use in setting? What are key factors that facilitated EWS’ successful use in setting? [Response time 7 minutes each]   *Model Facilitator writes on pre-populated blank sticky notes as participants share out, reflecting for understanding as she goes, if time is needed slow down reflection so theme builder can catch-up before share out. Theme builder send Teams message if more time is needed.*  *Theme Builder organizes cards thematically as she hears them.*  *Community Facilitator monitors chat for comments and notifies model facilitator when comments are posted. Theme Builder captures verbal comments and notifies facilitator.*  *Reflectors contact Model Facilitator by Teams chat to direct her understanding and probing.*  Model Facilitator: Calls in individuals who are speaking less toward the end. Hands off to Theme Builder. |
| 9:15-30  20 minutes | Theme Builder:  Reflectors: | **Share out Variable Elicitation by Theme**  *Community Facilitator navigates to Themes only in Miro (column 2)*  I will review these themes that I saw. Please let me know if you have any suggestions for any changes, including moving factors around or different themes.  Explain groupings and reasoning behind the groupings.  *Reflectors share feedback to theme builder by Teams chat.* |
| 9:30-45  15 minutes | Closer: | Today we started to identify facilitators and barriers to use of EWS at Duke Health. In our next session, we’ll explore some of the interconnections between these factors and how they manifest overtime.     - Directly after today’s session, you will receive an email link to share feedback on this session through a brief form. It should take you about 10-15 minutes to complete. We will also be in touch regarding your study compensation for today’s session. - We invite you to review the artifacts from our workshop today offline and provide your notes. You may have additional thoughts that you didn’t get to share today or weren’t quite ready, or you may have another idea after the time has passed. The more we hear from you the better to make sure that what we produce reflects your view. We will send a link to the whiteboard from today and instructions for posting your comments. Is Teams a good way for us to share things with this group (or another platform)? - Lastly, we will contact you soon to schedule for the next session, which will occur around late April/early May. We will be in touch with everyone individually about scheduling.   Thank you so much for your participation and great discussion today.  *Community Facilitator will send out feedback form via REDCap during this time.*    *All team members stay on call for short debrief*    *Recorder* *save Miro documents (screen shot, …), chat, Zoom recording.* |

# Detailed Agenda – Session 2: Graphs Over Time

| Time | **Lead(s)** | **Activity** |
| --- | --- | --- |
| Before session |  | **Set-up**   - pre-populated list of cleaned, most endorsed barriers / facilitators from the last session - pre-populated graph templates on the white board for ease of access - select variable to show how to do graphs over time - invitation to participants to access Miro (sent via email before session 2) |
| 15 minutes before session start | **All** | *Team members join call*   - Login to Miro & have both Voting and GOT boards open - Community Facilitator - open EWS PSD slides (slide 3), Miro board (team view) and facilitation manual (downloaded version to copy text for chat) - Open Teams app for backchannel communication |
| 5 minutes  8:00-8:05am | Community Facilitator: | **As people enter the Zoom**  Ask participants to change their Zoom labels to their first name. We will also share the slide deck in the chat – please click the file to download/ then open to reference during today’s session.  *Community Facilitator to verbally share below instructions and post instructions/share slide deck document in the chat****.***   - Hi and welcome! As you settle in, please add your name to your Zoom label (you can update this by clicking the 3 dots in the top right corner of your Zoom square then selecting ‘Rename’ at the bottom of the list) and open/login into Miro and navigate to today’s board called, Graphs Over Time – 5/4/23. - Share slide deck document in chat   Once everyone has entered Zoom: Hi everyone, thank you so much for joining. Before we begin, I wanted to let you know that we are starting our recording of today’s session now. Thank you.  ***Community Facilitator starts recording*** |
| 5 minutes  8:05-8:10am | Convener: | **Opening: Walk through tasks**  *Community Facilitator opens EWS PSD slides (slide 3) on shared screen, then show slide 4*  Convener:  Good morning.  Last time we met, we talked about the trajectory of EWS adoption.  We are looking at this from a health system perspective…  When decision to implement, high commitment, assume it’s good…    However, we have seen that commitment can wane as people begin to use the score in practice.    We want your help to identify variables that affect adoption.    We talked about these variables during the last workshop.  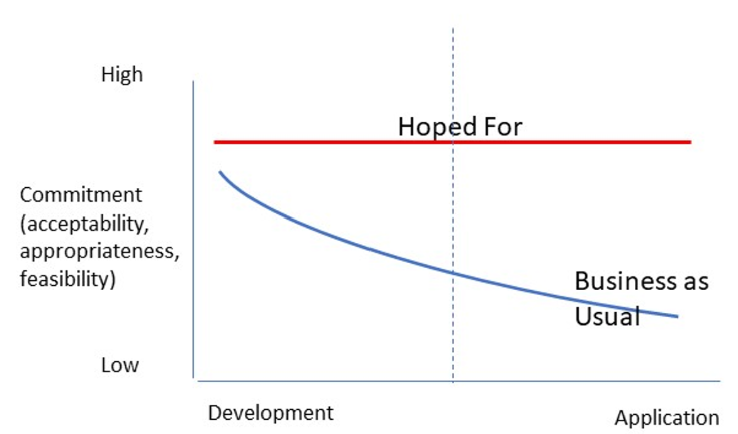  We have since consolidated them based on similar themes.  The point of this meeting is to get your feedback and to start thinking about *how* these variables *can change over time.*  We will be creating graphs for the consolidated themes last week*.*  *Plain language, down the line, what stage we are in the process...* |
| 60 minutes 8:10-9:10am |  | **Task ask: Graphs over Time**  *Community Facilitator opens Miro Graphs Over Time board (team view) on shared screen*  Facilitator shows all 10 variables.  These are the concepts extracted from the previous conversation. We may not have time to go over everything so we want to hear from you which ones resonate most with you.  Remind them that we want to think about variables that influence downstream adoption of EWS ---including design and workflow factors--- and sketch how they have changed over time.  5-7 minutes for each  Provide working definition.  Graph Over Time  Facilitator draws example GoT using one of the prior barriers/facilitator: *(Draw out in real time, explaining trajectories as goes along, Add clarifying points at completion)*   - Example of EHR changes post COVID decreasing accuracy - Label the x axis as time with a beginning and end time (e.g., 2018, COVID (dashed line), and present) - Label the y axis accuracy - Probes: Where should the line start? What happens after COVID? Where is is now? (line can take any form)   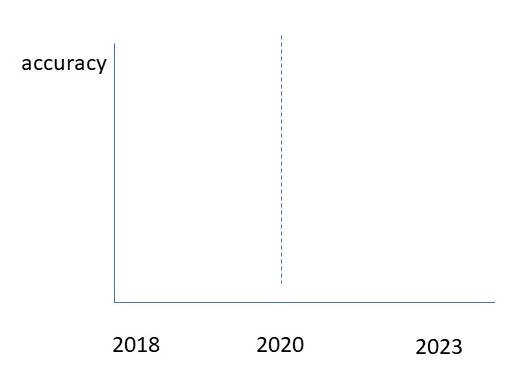  Reflect back what you heard and ask clarifying questions as needed.  Now let’s look at [Variable].  *Prompt: In thinking about [variable] how have you experienced or observed the variable changing? The time axis can also be qualitative (start to finish). If not clear, ask them about what should go on the x/time axis (e.g. years, development to application, within the patient interaction, over the course of the day).*  *Thinking about how [variable] influences adoption, would you want the trajectory to go up or down?*   - *Business as usual line (black)* - *Continuing from that line- diverge into a red line to represent feared changed, and a green line to show hoped for change.*   *Note to say it’s a vehicle for discussion not perfectionistic.* |
| 10 minutes 9:10-9:20am | Facilitator: | **Vote on variables**  *Community Facilitator closes out PowerPoint, moves to Miro voting board (team view)*  You may have to toggle your screen...Does everyone see this...  Please open the Miro board named EWS Voting Session - 4/21/23  Facilitator: Now we’re going to vote on the variables that we identified in our last session. If you have thoughts or comments while you are voting, please hold on to that for when we have discussion.  Please open the Miro board named EWS Voting Session - 4/21/23 You’ll see a voting pop up appear on your screen – click join voting to start. You will have a total of 7 votes and we will give you 2-3 minutes – to vote, click on the sticky notes. When you’ve used all of your votes, click done to submit. Community facilitator add these voting instructions in chat.  All team members take photo of voting results in Miro before facilitator moves to the graphs over time board. |
| 10 minutes 9:20-9:30am | Facilitator: | **Closing**  Facilitator: Here we started to explore how factors that influence and/or are influenced by CDS adoption change over time. The next session, *CLD, will explore some of the interconnections between those factors* that help us understand the trend over time.   - Directly after today’s session, you will receive an emailed link to share feedback on this session through a brief form. It should take you around 10 minutes to complete. We will also be in touch regarding your study compensation for today’s session and scheduling for the last session, which will occur around July (we can be flexible around summer travel plans) - We invite you to review the artifacts from our workshop today offline and provide your notes. You may have additional thoughts that you didn’t get to share today or weren’t quite ready, or you may have another idea after the time has passed. The more we hear from you the better to make sure that what we produce reflects your view. We’ll be in touch when those are ready to review.   Thank you so much for your participation and great discussion today.  *Community Facilitator will send out feedback form via REDCap during this time.*    *All team members stay on call for short debrief*  *Recorder* *save Miro documents (screen shot, …), chat, Zoom recording.* |

# Session 3: Causal Mapping

| 30 minutes | Facilitator: | **Causal Mapping in Small Groups**  *Draft Casual Loop Diagram to be pre-loaded on to Zoom whiteboard*  Hi and welcome! As you settle in, please add your name to your Zoom label (you can update this by clicking the 3 dots in the top right corner of your Zoom square then selecting ‘Rename’ at the bottom of the list) and open/login into Miro and navigate to today’s board called, TBD.  Once everyone has entered Zoom: Hi everyone, thank you so much for joining. Before we begin, I wanted to let you know that we are starting our recording of today’s session now. Thank you.  **Intro to Activity (5 min)**  Reminder of problem.  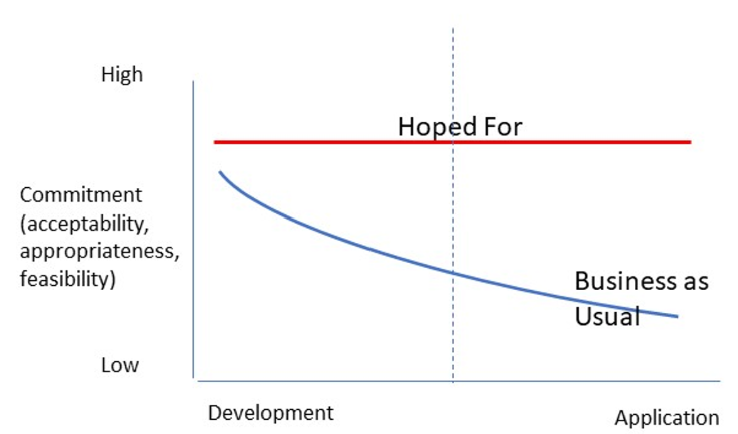  When decision to implement an EWS, commitment from a system perspective high during development phase- viewed as acceptable, appropriate, feasible. When put into practice, though, commitment waned. What happened?  We took what you said and put them into concepts. These concepts have relationships. We have come up with understanding of how they are interrelated.  Explain we want to explore the interconnections between variables that we have discussed to help us understand what affects EWS adoption over time. *Explain they will have the opportunity to refine / provide disagreements offline, so don’t focus too much on those components for the sake of time.*  We will use a visual way to show the interconnections. In this way, we will make what we each know from our experiences and views explicit.  Refer to CLD. We can tell a story from this diagram.  *Remind to correct for clarity understanding (content or misinterpretations, etc.)*  Let’s look at this diagram.  *Zoom in on the big loop.*  What it shows us is that there are two pathways leading to acceptability of the score, so whether nurses like it or not.  And these pathways come from accessibility.  The “s” means same direction, a positive relationship.  In one pathway, the more that you can access the score, the more able you are to actually see the score, and then you are more accepting.  In another pathway, the more accessible it is, the more you will be able to see and put in data in a timely manner, then the more accurate the score will be, and then you find it more appropriate for your workflow (for example, addressing the actual issue and not something that maybe had to do with their baseline), and then the more accepting you are.  And then what impacts accessibility is the number of patients needing checks (so the more patients you have to check on when you are on the floor, the more you are running around and not at the computer, and so the score is less accessible to you. The o means opposite direction or negative relationship.  Therefore, when the number of patients increases accessibility and then therefore timeliness may decrease.  Also, the less accurate the score is, the more the developers want the feedback, so they can recalibrate.  Let’s talk about these relationships. We want to know if this reflects what you said, your experience, or not. We can make changes including adding to it.  *Zoom in on accessibility and timeliness first, conduct prompts, then clarify each segment as we move through the loop for clarification*.  Prompts:   - Do these variables make sense or is there another one that you would use to explain the cause and effect? (For example, is it really number of patients needing checks that affects accessibility or is there something else like not enough staff?) Does this directionality seem correct?   *When someone makes a suggestion, include this in the drawing of the model in order to visualize what is meant. The facilitator needs to check with other group members if they agree with the proposed relation. If someone disagrees, the facilitator can ask for clarification and try to determine what the group thinks the relationship should be. Remind the group that if a discussion goes on too long, the group can choose to temporarily 'park' this item and continue with another part of the model.*  *When in doubt about including something, ask, If I were to double or halve this variable, would it have a significant effect on the issue I am mapping? If not, it probably can be omitted.*  *Ask them to explain what they mean so that we get detail.*   - Why does this happen? (*to uncover further upstream causality*) Is there something else going on? Does this relationship depend on something else? (the variable pairs and directionality) - What does this affect? (*to determine further downstream causality.*) Is there something else going on? Does this relationship depend on something else? (the variable pairs and directionality) - Does time matter here? Does this happen fast or slowly? Is there something that would delay this?   Make sure to iteratively do the following   - focus on clarifying instructions and providing reinforcement to the process itself - focus on helping individuals clarify their representations and modes - Validate and inquire for more information and confirm when placing on the map   Highlighting anything interesting that came up or lingering questions. Invite questions from the large group. |
| --- | --- | --- |
| Closing |  | Thank you for your input today. We will send you a clean version of this Causal Loop Model that we just created. Please feel free to reply if you have any other thoughts. Otherwise, the only other request we have is share feedback on this session.  Directly after today’s session, you will receive an emailed link to share feedback through a brief form. It should take you around 10 minutes to complete.  There is one question where we ask about concerns about the modeling process. By modeling process, we mean what we just did- the Causal Loop Model- not the algorithmic model.  We will also be in touch regarding your study compensation for today’s session.  *All team members stay on call for short debrief*  *Recorder* *save Miro documents (screen shot, …), chat, Zoom recording.*  Offline task: identify any areas they agree or disagree with and make note of suggestions to refine the model  **Offline follow-up & Next steps**  The modeler makes changes in a different color marker.    The modeler summarizes refinements made to the model, pointing out reinforcing and balancing loops, and highlights any emerging insights |
| After meeting/offline |  | **Reflector Feedback**  *Highlight system insights & mental model shifts* |
