# Supplementary material for: Engaging Hospital Staff to Identify Levers for Adoption of Clinical Decision Support: Protocol for a Single-Site Case Study Using System Dynamics Group Model Building
Source: JMIR Res Protoc. 2026 Apr 21;15:e80848. doi: 10.2196/80848 (PMC13099119; doi:10.2196/80848)
Supplement: Multimedia Appendix 2 [file resprot-v15-e80848-s002.doc]

**Engaging Multidisciplinary Health System Stakeholders to Create a Process for Implementing Machine-Learning Enabled Clinical Decision Support – Evaluation & Feedback**

**Workshop Facilitator:** **Date:**

| **Directions:** In rating today’s workshop, please respond to each item thoughtfully. We will incorporate your suggestions and make improvements. | Excellent | Very Good | Good | Fair | Poor | Very Poor | Doesn’t Apply |
| --- | --- | --- | --- | --- | --- | --- | --- |
| 1. The facilitator’s overall effectiveness was: | 6 | 5 | 4 | 3 | 2 | 1 | N/A |
| 1. The online environment was | 6 | 5 | 4 | 3 | 2 | 1 | N/A |
| 1. The potential of modeling to improve decision-making and procedures in the clinic *by including all staff and getting staff on the same page* (as compared to decision-making without modeling) is… | 6 | 5 | 4 | 3 | 2 | 1 | N/A |
| 1. The potential of the system dynamics modeling process to help me accomplish my job responsibilities is… | 6 | 5 | 4 | 3 | 2 | 1 | N/A |
| 1. Please select the ways that you would prefer to provide model input and feedback (select all that apply and/or make suggestions below): | Staff Meeting | CDS Stakeholder group | One-on-one meeting | Email | Other | Unsure |  |
| 1. My biggest concern about the modeling process is… |  | | | | | | |
| 1. I believe the most useful aspect of the modeling process is likely to be… |  | | | | | | |

***Other anonymous comments/feedback you would like share:***
