# Supplementary material for: Engaging Hospital Staff to Identify Levers for Adoption of Clinical Decision Support: Protocol for a Single-Site Case Study Using System Dynamics Group Model Building
Source: JMIR Res Protoc. 2026 Apr 21;15:e80848. doi: 10.2196/80848 (PMC13099119; doi:10.2196/80848)
Supplement: Multimedia Appendix 3 [file resprot-v15-e80848-s003.docx]

**AI Use in Manuscript Preparation**

The manuscript was initially drafted entirely by the authors without AI assistance. Generative AI (OpenAI’s ChatGPT, GPT-5) was subsequently used solely to clarify text, improve flow, and ensure consistency throughout the manuscript. AI-generated suggestions were limited to wording, transitions, and formatting; all scientific content, study design, and interpretation were determined solely by the authors. All AI-generated text was reviewed, verified, and edited by the authors for accuracy, completeness, and alignment with the intended meaning.

To support transparency, we have included a table of prompts used during AI-assisted text refinement, along with the corresponding sections of the manuscript where AI input was applied.

| **Prompt** | **Section Where AI Was Used** | **Purpose** |
| --- | --- | --- |
| “Update the abstract to make it consistent with the revised paper” | Abstract | Clarify wording and ensure alignment with main text edits |
| “Suggest transitional phrases for Methods section” | Methods | Improve flow and readability |
| “Make the Methods description of workshops consistent with later edits” | Methods – Group Model Building | Clarify sequence and structure of sessions |
| “Edit Results in abstract to match main text description” | Abstract | Ensure consistency between abstract and main text |
| “Clarify use of AI in manuscript for transparency” | Methods – AI disclosure | Draft AI use statement for Methods section |
